# Bibliography

**Published:** 1900-04

**Authors:** 


### Bibliography.

Gould's Pocket Pronouncing Dictionary. Fourth Revised Edition. By George M. Gould, A.M., M.D. P. Blakiston's Son & Co., Philadelphia, 1900.

Dr. Gould's achievements in dictionary making are excellent. That one hundred thousand copies had been sold at the time of the appearance of the last edition is abundant evidence of the appreciation of his work. The new edition is invaluable to students of medicine, dentistry, and pharmacy.

It is enlarged by the addition of nine thousand words and their definitions, largely made up of eponymic clinical terms. The Dose Table has also been enlarged by the inclusion of the names and doses of drugs recently introduced, and the whole work has been carefully edited. Much credit is also due the publishers and printers for their part in the production of this work.

